# Factors influencing adherence to an app-based exercise program in adolescents with painful hyperkyphosis

**DOI:** 10.1186/s13013-018-0159-x

**Published:** 2018-07-18

**Authors:** Karina A. Zapata, Sharon S. Wang-Price, Tina S. Fletcher, Charles E. Johnston

**Affiliations:** 1 Therapy Services, Texas Scottish Rite Hospital for Children, 2222 Welborn Street, Dallas, TX 75219 USA; 2 School of Physical Therapy, Texas Woman’s University–Dallas, Dallas, TX USA; 3 School of Occupational Therapy, Texas Woman’s University–Dallas, Dallas, TX USA; 4 Texas Scottish Rite Hospital for Children, Dallas, TX USA

## Abstract

**Background:**

Software applications (apps) could potentially promote exercise adherence. However, it is unclear whether adolescents with painful hyperkyphosis will use an app designed for a home exercise program. The purpose of this study is to assess factors regarding adherence to an app-based home exercise program in adolescents with hyperkyphosis and back pain who were provided a one-time exercise treatment.

**Methods:**

Twenty-one participants were instructed in a one-time exercise treatment and asked to complete a home exercise program 3 times a week for 6 months using an app called PT PAL. At a 6-month follow-up, 14 participants completed a survey assessing factors related to their experiences using the app and their treatment engagement.

**Results:**

Although most participants did not use the app, they reported performing their exercises a few times per week. The adolescent participants considered the app to be more of a barrier than a supportive measure for promoting exercise adherence. Most participants still reported bothersome back pain.

**Conclusions:**

Although adherence to the 6-month app-based home exercise program was not successful, adolescents still viewed technology support such as text reminders as a potential solution.

**Trial registration:**

ClinicalTrials.gov Identifier: NCT03212664. Registered 11 July 2017. Retrospectively registered.

## Background

Adolescents with hyperkyphosis have decreased quality-of-life (QOL), particularly the self-image and appearance components [1, 2]. Hyperkyphosis is also associated with back pain in long-term follow-up studies [3, 4]. The standard medical management for kyphosis includes observation with or without exercises, bracing, and surgery. In North America, utilization of physical therapy (PT) for kyphosis varies by institution, and the optimal treatment is controversial. Although PT exercises designed to improve pain and posture are generally recommended for hyperkyphosis, the evidence for the effectiveness of these exercises is very limited [5, 6].

Exercise adherence is problematic in the rehabilitation of adolescents with back pain [7–9]. Numerous factors influence adherence to an exercise program such as personal characteristics, treatment and disease variables, and the patient-to-physical therapist interaction [7, 10]. Specifically, personal characteristics related to patients’ behaviors, including enjoyment of the activity, social support, and self-motivation could have a profound impact on exercise adherence [7, 10]. Of these personal characteristics, motivation can be influenced by a patient’s self-esteem or perceived competence [7]. Treatment variables, such as the complexity of exercises, may also be factors [10]. Additionally, chronic illnesses and pain experiences are examples of disease variables that can influence exercise adherence [10]. Lastly, exercise adherence factors related to the patient-to-physical therapist interaction include patients’ need for more feedback, specifically positive feedback [7, 10].

Software applications (apps) have the potential to play an important role in promoting exercise adherence. Apps can monitor patients remotely, are cheap, can provide reminders, and can enable feedback to patients. Although apps are widely used by adolescents, app-based exercise programs have not been incorporated in rehabilitation for adolescents with musculoskeletal disorders [11]. In addition, a systematic review showed limited evidence regarding the effectiveness of using apps to increase physical activity in adolescents [8]. Furthermore, apps aimed at increasing physical activity in adolescents were not effective [12]. Although app-based exercise programs can provide objective information regarding exercise adherence, it is unclear whether adolescents with painful hyperkyphosis will use an app designed for a home exercise program (HEP). Therefore, the purpose of this study was to assess factors regarding adherence to an app-based exercise program in adolescents with hyperkyphosis and back pain after one-time exercise treatment followed by a 6-month app-based HEP.

## Methods

### Participants

This study was a prospective pre-post study design. This study was approved by the Institutional Review Board of the primary investigator’s affiliated institute and registered with ClinicalTrials.gov. Participants were recruited from a tertiary facility that holds specialty clinics, including spine deformity, and is staffed by a multidisciplinary team including physical therapists. Prior to data collection, each adolescent participant’s assent and caregiver’s consent to participate in the study were obtained. Eligible participants were adolescents with hyperkyphosis, including Scheuermann’s kyphosis, and met the following inclusion criteria: ages 10 to 18 years, Cobb angles at least 50°, and pain > 2 on the numeric pain rating scale (NPRS) during the past week. Participants’ pain intensity was evaluated according to the NPRS on a scale of 0 (no pain) to 10 (worst imaginable pain) [13]. Exclusion criteria included scoliosis greater than 25° Cobb angle, conditions preventing understanding and compliance with an exercise schedule, current brace wear, previous spine surgery, and inability to commit to at least 15 min of exercises for 3 days a week for 6 months.

Once eligibility was determined, each participant was asked to complete the Scoliosis Research Society-22 Health-Related Quality-of-Life Questionnaire (SRS-22r) to evaluate participants’ QOL. The SRS-22r includes five domains: pain, self-image/appearance, function/activity, mental health, and management satisfaction. Higher scores indicate better scoliosis-related QOL. The SRS-22r is reliable and valid in adolescent idiopathic scoliosis (AIS) [14]. Although it has not been validated for assessing adolescent patients with hyperkyphosis, it is considered well suited [1, 2]. Participants were also asked about their physical activity level, which was defined as the number of hours of organized physical activity per week.

Next, a physical therapist instructed each participant in PT exercise treatment targeting spinal stabilization. The standard PT practice at the investigators’ facility is to provide one-time exercise instruction on the same day that patients are seen by the orthopedic surgeon. Patients also typically prefer this treatment model, as it is less burdensome and more cost-effective, especially because many patients live at least an hour from our facility and come from low socioeconomic backgrounds, thus having difficulty returning for PT on a regular basis. Participants were instructed to perform each exercise for 100 s based on a previous work [9]. Instructions included performing a total of 5 exercises targeting the trunk and core for at least 3 days a week for 6 months. Participants were also asked to refrain from slouching while sitting throughout the day.

In addition, each participant was given access to a software app called Pt Pal (Los Angeles, CA) on their smart phone or tablet. Pt Pal uses a cloud-based platform which sends patients (children or adults) their exercises and exercise prescriptions digitally. The physical therapist can use a web portal to add participants and enter their exercises independently, and the participants can access their prescribed exercises from the app on their phone or tablet. After participants log in to the app, they see their prescribed exercises by image and exercise name. Exercise pictures are in black-and-white. To perform an exercise, they click on the respective exercise, which shows the same picture and written instructions on how to perform the exercise. The prescribed amount of time counts down similar to an interval timer. During the time of this study, Pt Pal did not have the ability to send reminders to participants to do their exercises and to allow participants to give feedback. In addition, exercises adherence was defined by examining each participant’s app and averaging the percentage of exercise sessions completed out of 78 sessions (3 sessions per week for 6 months).

Participants were asked to use the app whenever they performed their exercises throughout the 6-month period. The principal investigator (PI), a physical therapist, had administrative access to the app to monitor exercise adherence. Each participant was contacted and instructed to use the app within 2 weeks after study initiation in an attempt to maximize exercise adherence. During this follow-up contact, the PI answered questions regarding their exercises or app use if there was any. In addition, participants were instructed to contact the PI when they had additional questions regarding their exercises or the app. However, most participants still did not use the app. Consequently, the effectiveness of the app on exercise adherence was unable to be assessed. In order to understand the adolescent participants’ lack of interest in using the app for their HEP, the investigators developed a survey to obtain information about these participants’ experiences using the app and performing the exercises.

### Instrumentation: survey

The PI administered a 19-item survey to all participants either in person at a 6-month follow-up or over the phone if they did not return for follow-up. The PI read the survey to all participants to ensure their understanding of the instructions and the survey, and wrote down responses to gather details related to their experiences with the app and exercises.

**Fig. 1:**
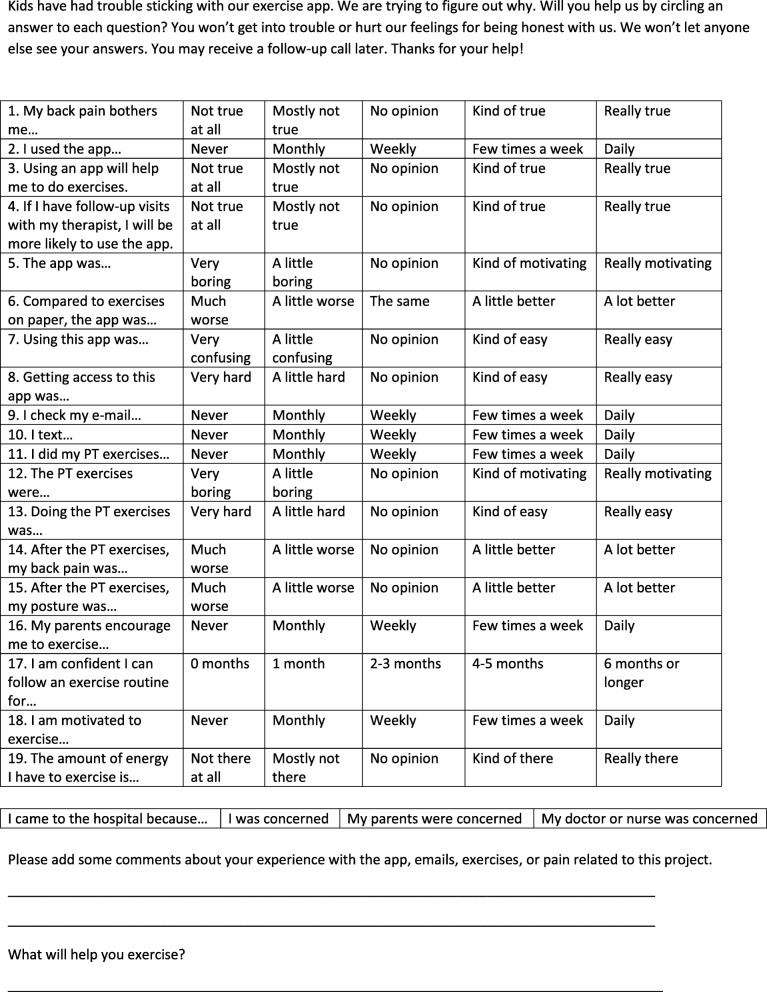
Survey

The investigators developed the survey (Fig. 1) based on the previous works that identified factors related to exercise adherence, app usability, and acceptability [7, 10, 12]. Each question was scored on a scale of 1 to 5, with 1 indicating the smallest endorsement or most disagreeable response, and 5 indicating the greatest endorsement or most agreeable response (e.g., better, more agreement, easier). The survey included factors such as motivation, support, the environment, and reinforcing value [7]. In particular, personal characteristics, disease variables, treatment variables, and the patient-to-physical therapist interaction were considered [8, 10, 15–17]. The questions were aimed at understanding participants’ views regarding the exercises provided, exercising in general, their back pain, the app, and technological preferences. Two additional open-ended questions offered the participants opportunities to provide more explanation or details (see Fig. 1).

Prior to implementing the survey, six healthy adolescents without any deformity or back pain reviewed the questions to ensure that wording was appropriate for an adolescent patient population. Specifically, reviewers provided feedback to include questions should not appear punitive (e.g., “They need to know they aren’t in trouble”), questions should not be similar in tone or formatting to formal testing protocols (e.g., “Don’t use Scantron forms or punch cards”), and the survey should be easy to complete (e.g., “Provide some lines, not too many though”).

### Data analysis

Means, standard deviations, and paired *t* tests (*P* < 0.05) were used to describe all the survey variables. Two investigators independently analyzed responses to the two open-ended questions. Each response, whether a word, phrase, or sentence, was analyzed to determine whether it contained enough information to become a unit of analysis or node. To ensure rigor of this study, each investigator independently eliminated extraneous words, then created node sets from responses. This resulted in 54 nodes for the request for participants to provide any additional comments, and 14 nodes for “What will help you exercise?” The investigators then independently grouped nodes into agreed-upon categories. In the final phase of analysis, the investigators collaborated to create one categorized data set.

## Results

Twenty-one participants were enrolled in the original study, but only 14 either returned for the 6-month post-treatment assessment or responded to phone calls to complete the survey. See Table 1 for participant characteristics at baseline. Participants who were lost to follow-up did not differ by age, BMI, physical activity, curve magnitude, pain intensity, and SRS-22 scores as compared to the participants who completed the survey at the 6-month follow-up (*P* > 0.05). Participants had intermittent back pain for about 2 years and 5 months on average. The participants were mostly sedentary, averaging 1.3 h a week of physical activity at baseline. Lastly, the SRS-22 scores did not significantly improve at the 6-month follow-up (Table 2).

**Table 1:** Characteristics of participants (*n* = 14)

	Pretest
Age (years)*	15.3 ± 2.0
Gender	5 girls
	9 boys
Ethnicity	11 Caucasian
	3 Hispanic
Body mass index (kg/m^2^)*	27.0 ± 0.6
Physical activity (hrs/wk)*	1.3 ± 0.2
Risser grade*	3.3 ± 1.7
Curve magnitude*	60.1 ± 0.9°
Days of back pain*	870 ± 64
Pain intensity*	5.2 ± 2.1

*Data are mean ± SD

**Table 2:** Average scores and standard deviations (SD) of the Scoliosis Research Society-22 Health-Related Quality-of-Life Questionnaire (SRS-22r) at pretest (*n* = 14) and at posttest (*n* = 14)

Outcome measure	Pretest	Posttest	*P* value
SRS-22r total	3.3 ± 0.3	3.6 ± 0.4	0.09
Pain	3.2 ± 0.6	3.5 ± 1.0	0.40
Self-image	2.9 ± 0.8	3.1 ± 0.9	0.34
Function	3.9 ± 0.6	4.2 ± 0.9	0.13
Mental Health	3.5 ± 0.9	3.7 ± 0.4	0.13
Satisfaction	3.5 ± 0.9	3.5 ± 0.9	0.39

The app usage reports revealed that most participants did not use the app. One participant did not have a Smart phone or tablet. This participant logged exercise adherence on a sheet of paper, which was used to assess exercise adherence, and we could not assess his adherence according to the app. One participant had 100% HEP adherence according to the app usage report. The remaining 12 participants either did not use the app or used it less than once per week. This app usage report was similar to participants’ response to question 2 of the survey regarding the app usage, for which most participants indicated that they used the app less than weekly (question 2, Table 3).

**Table 3:** Means and standard deviations of survey responses

Question	Mean ± SD* (*n*)	Means defined**
1. My back pain bothers me…	3.9 ± 1.6 (*n* = 14)	Kind of true
2. I used the app…	2.6 ± 1.2 (*n* = 14)	Weekly
3. Using an app will help me to do exercises.	3.7 ± 1.1 (*n* = 14)	Kind of true
4. If I have follow-up visits with my therapist, I will be more likely to use the app.	3.5 ± 1.5 (*n* = 14)	Kind of true
5. The app was…	3.0 ± .6 (*n* = 11)	No opinion
6. Compared to exercises on paper, the app was…	3.8 ± .8 (*n* = 12)	A little better
7. Using this app was…	3.4 ± 1.2 (*n* = 12)	No opinion
8. Getting access to this app was…	3.1 ± 1.7 (*n* = 13)	No opinion
9. I check my e-mail…	3.6 ± 1.3 (*n* = 14)	Few times a week
10. I text…	4.7 ± .8 (*n* = 14)	Daily
11. I did my PT exercises…	3.6 ± .9 (*n* = 14)	Few times a week
12. The PT exercises were…	2.8 ± .7 (*n* = 14)	No opinion
13. Doing the PT exercises was…	3.6 ± 1.2 (*n* = 14)	Kind of easy
14. After the PT exercises, my back pain was…	3.4 ± 1.1 (*n* = 14)	No opinion
15. After the PT exercises, my posture was…	3.4 ± .7 (*n* = 14)	No opinion
16. My parents encourage me to exercise…	3.6 ± 1.2 (*n* = 14)	Few times a week
17. I am confident I can follow an exercise routine for…	3.9 ± 1.5 (*n* = 14)	4–5 months
18. I am motivated to exercise…	3.8 ± 1.1 (*n* = 14)	Few times a week
19. The amount of energy I have to exercise is…	3.6 ± 1.4 (*n* = 14)	Kind of there

*Scale of 1 to 5. *1* smallest endorsement or most disagreeable response, *5* greatest endorsement or most agreeable response

**Means defined according to rounding rules

Participants were largely ambivalent regarding their attitudes regarding the app (questions 3–8, Fig. 1) and regarding the exercises (questions 12–15, Table 3). When asked for more details about the app, participants cited having technical difficulties such as logging in, having limited storage on their phone, and forgetting to use the app when they performed their exercises. Participants suggested that receiving reminders such as text messages or pop-up notifications to use the app might facilitate app use. In addition, participants indicated that they texted almost daily, significantly more than the frequency of checking their e-mail (questions 9–10, Table 3).

Although most participants did not use the app, 10 participants reported that they performed their exercises a few times per week (question 11, Table 3). Nine of them reported that they were motivated to exercise at least a few times a week, and that they were confident that they could follow an exercise routine for at least 4 months (questions 17–18, Table 3). When asked what would help them to perform a HEP, a specific goal like training for marching band was mentioned. Participants also reported that adherence to an exercise program would likely increase if their parents were involved in their HEP or if the app provided exercise reminders. Other responses included that the exercise had to be fun (1 response) and decrease pain (2 responses). However, three participants were not sure what would help them exercise.

Surprisingly, only three participants who reported no back pain at follow-up attributed the exercises to helping their back pain. The remaining 11 participants still reported having back pain and that their back pain bothered them (question 1, Table 3). Eight of these 11 participants reported that the exercises did not help their back pain at all. Although the other 3 of these 11 participants reported that their back pain still bothered them, they considered that the exercises had helped their back pain.

**Fig. 2:**
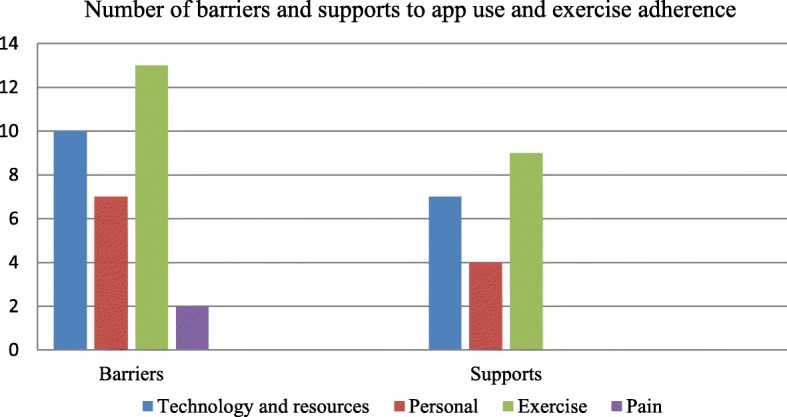
Numbers of barriers and supports to app use and exercise adherence

**Fig. 3:**
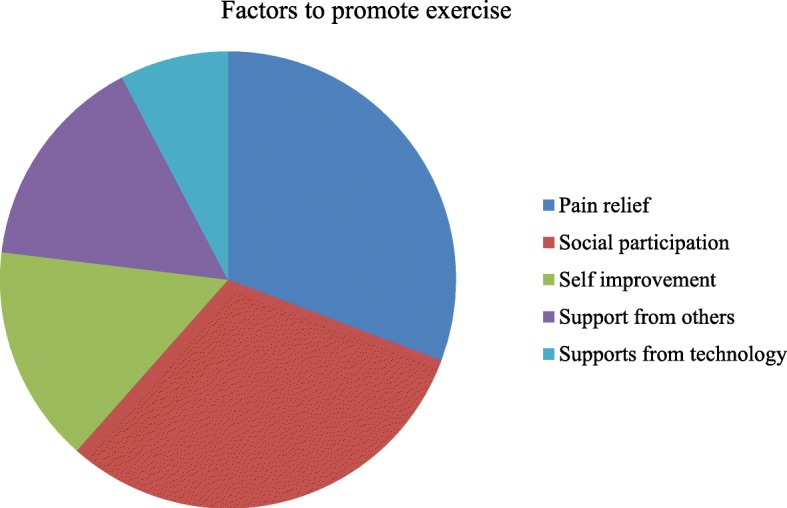
Factors to promote exercise. List of abbreviations: quality-of-life (QOL), physical therapy (PT), applications (apps), home exercise program (HEP), numeric pain rating scale (NPRS), Scoliosis Research Society-22 Health-Related Quality-of-Life Questionnaire (SRS-22r), adolescent idiopathic scoliosis (AIS)

Results from the query for additional comments fell into either barriers (32) or supports (20) for app use and exercise adherence (Fig. 2). Barriers included technology and resources such as technology device limitations, connectivity, and Internet access; personal factors such as forgetfulness, poor posture, and pregnancy; or unchanged or worsening pain. Supportive measures included technology and resources such as help from people (e.g., mother and physical therapist) and equipment (e.g., exercise band and foam roll); personal attributes such as happy disposition, habit of daily exercise, and owning a phone; and exercise belief such as the exercises could cause pain reduction and muscle strengthening (Fig. 2). Further, results from the query asking participants what would help them exercise included pain relief (4 responses), social participation (4 responses, e.g., marching in a band, having fun with friends, and playing basketball), self-improvement (2 responses), receiving support from others (2 responses), or receiving supports from technology devices (1 response) (Fig. 3).

## Discussion

A variety of factors contributed to the lack of app use in adolescents with painful hyperkyphosis. Technology-related, connectivity-related, and participant-induced considerations all contributed to app adherence. When the free-text questions were analyzed, adolescents considered that using an app for exercise adherence was more of a barrier than a supportive measure. Specifically, technology and resources were more frequently viewed by the participants as barriers than as supportive measures. Several participants mentioned technical and logistical difficulties using the app, and app-specific troubleshooting may have been beneficial to improve the app’s usability. Similarly, a study which used a mobile phone app to monitor caloric intake found that only 20% of participants consistently used an app, namely, those with higher exercise motivation scores [18]. However, we did not explore participants’ initial willingness to use an app-based routine. Therefore, the preferences and feedback from the target users should be included in the early development of an app for its usage. In addition, because the app’s look and features may be more important to children than to adults, feedback form children regarding this aspect also should be included in the early stage of an app development. As some participants forgot to use the app, additional social media interaction such as daily text message reminders and individualized feedback may increase motivation for this patient population to perform prescribed exercises and use the app. Perhaps the physical therapist should collaborate with adolescents to determine which method of communication for a home program support would benefit them. Because many adolescents prefer texting, apps may be more beneficial for exercise adherence if text messages or pop-up alerts are incorporated in the app as another mechanism for therapists to provide feedback.

The most common rationale for participants to perform exercise was social participation and pain relief. Therefore, social participation should be included when designing exercises to improve exercise adherence for this patient population. Social environment, such as parents, friends, neighbors, teachers, and coaches may influence exercise adherence [19]. A systematic review also found strong evidence that multicomponent interventions involving the family or community could increase physical activity in adolescents, although studies referenced were school-based [8]. School social connections have been found to be a more important factor in increasing adolescents’ physical activity than the school physical environment [20]. One example of this can be illustrated by a participant who was motivated to exercise so that he could participate in marching band. Team building interventions also have improved exercise adherence and satisfaction in an adolescent exercise setting [21]. Another systematic review found an association between higher levels of physical activity among friends and higher levels of an individual’s physical activity [22]. However, there were mixed results regarding a friend’s sedentary behavior and individual sedentary behavior [22].

A family-centered approach could also align personal and technical supportive measures with patients’ capabilities and preferences to increase app use and exercise adherence. Strong evidence indicates that poor social or family support is a barrier for treatment adherence [16]. Involving families and social support systems is of particular importance to this patient population given their lower-than-normal QOL scores according to the SRS-22r [23–25]. The low SRS-22r scores indicated that the participants in this study had high-perceived back pain, low self-image, low physical function, and low mental health. Strong evidence has shown that psychological barriers such as depression, anxiety, and stress are barriers for treatment adherence [16].

Some researchers believe that decreased QOL in patients with hyperkyphosis implies increased indications for surgery [26]. However, non-operative management such as PT and psychology has not been adequately explored. We believe that patients with painful hyperkyphosis will benefit from more detailed psychological construct analysis. A more comprehensive treatment approach may also be necessary to address the chronic pain, sedentary lifestyles, obesity, and poor QOL scores that accompany painful hyperkyphosis, as evidence has shown that an intensive inpatient rehabilitation consisting of posture education, psychology, PT exercises, acupuncture, and the rare use of medication improved pain and posture in patients with Scheuermann kyphosis [27, 28].

More frequent in-person follow-ups by physical therapists and psychologists may help to promote behavior-changing strategies and help to evaluate whether an app for exercise may be an appropriate treatment for this patient population. Although most patients reported exercising a few times a week and that they were motivated to exercise to decrease back pain, we cannot be sure that these were not socially desirable answers, especially given that most of the patients were sedentary. Emphasizing physical activity in the context of social participation, a factor found to promote exercise, may be more enjoyable than a formal exercise program.

Although participants were ambivalent regarding whether they would be more likely to use the app if they had follow-up visits with their therapist, we did not objectively evaluate this assertion, as the participants only received a one-time supervised PT treatment only. In addition, the survey administration occurred at the 6-month follow-up with the orthopedic surgeon. Waiting 6 months may have influenced the validity of reported barriers. Earlier and multiple survey administrations may have been preferable so that problems with exercise adherence could be detected earlier. A previous study on adolescents with idiopathic scoliosis and back pain compared patients who were provided a one-time exercise treatment followed by an unsupervised 4-month HEP to patients who received 2 months of weekly supervised PT at 6-month follow-up [29]. Results showed that patients in the supervised PT group had greater improvements in pain and satisfaction compared to the one-time exercise treatment group [29]. Although patients from the above-mentioned study had scoliosis, the increased attention and social support received by patients in the supervised group may improve treatment adherence in patients with hyperkyphosis.

One study limitation is the small sample size, which is primarily due to the low return rate of participants at the 6-month follow-up despite efforts to phone and include those who did not return for their follow-up visit. Another study limitation is that we used a standard outcome measure specific to scoliosis rather than one for hyperkyphosis because no standardized outcome measures are available for this patient population. Therefore, the SRS-22r scores may not give a full representation of quality-of-life for the participants of this study. Finally, because most participants did not use the app, we had to rely on self-report measures of exercise adherence, thus introducing the potential for bias.

Future research should focus on examining strategies to improve exercise adherence to an app in adolescents with back pain caused by spinal disorders. Three factors: technology related, connectivity related, and participant induced, all contribute to app adherence and should each be more systematically evaluated so that their unique contribution to connected health therapy programming can be more strategically planned and realistically managed. In addition, because adolescents text frequently, future research should consider using Health Insurance Portability and Accountability Act (HIPAA)-secure text messages instead of e-mails or phone calls as a means of communication for follow-up needs. Finally, kyphosis-specific outcome measures should be developed, as current outcome measures evaluating quality-of-life (SRS-22r) and self-image (Spinal Appearance Questionnaire) have been validated in the AIS population and not in patients with kyphosis [14, 30].

## Conclusion

Patients with painful hyperkyphosis who were given a one-time exercise treatment followed by a 6-month app-based HEP did not use the app due to ambivalence regarding the usability and acceptability of the app and the exercises. Understanding a specific population’s technology habits is important before assuming potential effectiveness of an app or other electronic measure. Adolescents viewed technology support such as text reminders as a potential solution. A more comprehensive approach with additional research is needed to better understand how to improve app use to promote a successful rehabilitation for this patient population.
